# From Peer Victimization to Depressive Symptoms Through Acculturative Stress in South Korean Multicultural Adolescents: The Complex Moderating Roles of Family Support

**DOI:** 10.3390/children13081052

**Published:** 2026-08-06

**Authors:** Yangmi Lim

**Affiliations:** Department of Home Economics Education, College of Education, Jeonju University, Jeonju 55069, Republic of Korea; ym68@jj.ac.kr; Tel.: +82-63-220-2338

**Keywords:** peer victimization, acculturative stress, depressive symptoms, family support, multicultural adolescents, minority stress theory, moderated mediation

## Abstract

**Highlights:**

**What are the main findings?**

- Peer victimization in early middle school predicted higher depressive symptoms one year later, with a significant indirect association through acculturative stress among South Korean multicultural adolescents.
- Family support was not uniformly protective: it weakened the link from peer victimization to acculturative stress but strengthened the link from acculturative stress to depressive symptoms.

**What are the implications of the main findings?**

- Anti-bullying efforts for multicultural adolescents should recognize peer victimization as an external stressor associated with acculturative stress and depressive symptoms.
- Effective responses require coordinated school–family action: multicultural education, anti-bullying initiatives, targeted counseling for acculturative stress and depressive symptoms, and family guidance to promote constructive coping.

**Abstract:**

**Background/Objectives**: Adolescents from multicultural families in South Korea, most with foreign-born mothers and Korean fathers, may be vulnerable to peer victimization due to cultural, linguistic, or appearance-related differences. Grounded in minority stress theory, this study examined whether acculturative stress mediated the prospective association between peer victimization and depressive symptoms and whether family support, perceived across T1 (Year 1 of middle school) and T2 (Year 2 of middle school), moderated these pathways among adolescents from multicultural families. **Methods**: The analytic sample comprised 1218 adolescents (50.8% girls) drawn from the Multicultural Adolescents Panel Study, a national longitudinal dataset from South Korea. Mediation and moderated mediation analyses were conducted using the PROCESS macro. **Results**: Peer victimization at T1 was associated with higher depressive symptoms at T2 through acculturative stress assessed concurrently at T2, warranting caution in interpreting the indirect association as causal. Family support moderated the peer victimization–acculturative stress and acculturative stress–depressive symptoms paths, but not the direct peer victimization–depressive symptoms path. Specifically, family support weakened the association between peer victimization and acculturative stress, whereas it strengthened the association between acculturative stress and depressive symptoms. No overall moderated mediation pattern was evident because the two path-specific moderation effects operated in opposing directions and offset each other. **Conclusions**: These findings suggest that family support may operate differently across stress pathways. This study underscores the importance of culturally responsive school–family collaboration to reduce peer victimization, address acculturative stress and depressive symptoms, and equip families to support adolescents’ constructive coping.

## 1. Introduction

Traditionally considered monocultural, South Korea has undergone substantial cultural diversification through international marriage, global interconnectedness, and the worldwide spread of Korean popular culture [1]. Marriages involving a foreign spouse now account for approximately 10% of all marriages, most commonly between South Korean men and foreign-born women [2,3]. Since the 2008 enactment of the Multicultural Families Support Act, the term *multicultural family* has been widely institutionalized in policy, practice, and public discourse [1]. This diversification is increasingly visible in South Korean schools. Although total enrollment in elementary, middle, and high schools declined from 6,721,176 in 2012 to 5,028,775 in 2025, the number of students from multicultural families increased from 46,954 to 202,208 during the same period [4,5]. Most students from multicultural families were born in South Korea (91.5%), and the most common countries of origin of their foreign-born parents were Vietnam, China, and the Philippines [4]. These contrasting trends highlight the growing importance of understanding these students’ peer experiences, acculturative challenges, and psychological well-being.

The first year of middle school may be an especially challenging period for adolescents from multicultural families. Early adolescence involves rapid biological and socio-emotional development, while the transition to middle school requires adaptation to new routines, broader peer networks, and heightened academic expectations [6]. In South Korea, strong academic competition and peer comparison may further increase psychosocial risk. Depressive symptoms are therefore an important concern; in 2025, 25.7% of South Korean adolescents reported depressive symptoms [7].

Adolescents from multicultural families may also face peer and family stressors associated with depressive symptoms. Prior studies have documented bullying, relational victimization, and mistreatment related to language, appearance, skin color, or cultural background among adolescents from multicultural families; these experiences are associated with poorer psychological and school adjustment [8,9,10]. At home, foreign-born parents—often mothers—may have difficulty supporting adolescents’ school adjustment or communicating with schools because of limited familiarity with the Korean education system or language barriers [8,9]. These findings suggest that depressive symptoms among adolescents from multicultural families may be linked to psychological processes associated with their multicultural backgrounds. At the same time, more than half of multicultural adolescents who experienced school violence reported it to parents or other family members [10]. Accordingly, it is important to clarify how peer victimization is linked to depressive symptoms and whether family support shapes these pathways. Guided by minority stress theory [11], this study examines acculturative stress as a mediating mechanism linking peer victimization to depressive symptoms and family support as a moderator of these pathways among adolescents from multicultural families.

### 1.1. Theoretical Background: Minority Stress Theory

Proposed by Meyer [11], minority stress theory posits that chronic stressors associated with minority status contribute to elevated psychological distress and poor health outcomes. Defined by its origin in prejudice and social exclusion, minority stress is distinct from general forms of stress experienced by the broader population [12]. Minority stressors are often conceptualized as distal or proximal. Distal stressors are typically external and objectively observable events or conditions arising in the social environment, ranging from interpersonal victimization and discriminatory treatment to structural disadvantages. Proximal stress involves an individual’s internalized responses to such external pressures, encompassing both the cognitive appraisal of distal stressors as minority-related threats and the subsequent cognitive–affective responses, including expectations of rejection and internalized stigma [12,13]. Health outcomes include broader psychological or physical consequences, such as depressive and somatic symptoms. Distal stressors are theorized to contribute to proximal stress processes, which can operate as mediating mechanisms linking distal stressors with health outcomes among minority groups [12]. This theory also highlights the role of social support and other protective resources in buffering the health risks associated with minority stressors [14].

Originally proposed to address the lived experiences of sexual minorities, minority stress theory has since been extended to include diverse marginalized groups [12]. The adolescents examined in this study are from multicultural families and may therefore be conceptualized as members of a culturally minoritized group. Within the present study, peer victimization was conceptualized as a distal interpersonal stressor because it originates in the peer environment. Among adolescents from multicultural families, peer victimization may heighten acculturative stress, particularly when such experiences are perceived as being related to their multicultural backgrounds or minority status. These perceptions may intensify broader culturally specific strain related to perceived language difficulties, pressure to conform to Korean norms, cultural teasing, and mistreatment of adolescents or their families. Acculturative stress was therefore conceptualized as a proximal stress process because it reflects both adolescents’ cognitive appraisals of social and cultural experiences and the cognitive–affective responses arising from such appraisals. Acculturative stress may, in turn, contribute to depressive symptoms, including diminished interest, low energy, and depressed mood. Thus, acculturative stress reflects culturally specific stress related to minority experiences, whereas depressive symptoms represent broader, non-culture-specific psychological distress.

### 1.2. Peer Victimization and Depressive Symptoms in Adolescents from Multicultural Families

During early adolescence, youth become increasingly sensitive to social evaluation, face peer-related challenges, and seek a sense of group belonging [15]. Peer victimization may therefore be closely associated with depressive symptoms. Bullying, a common form of peer victimization, involves repeated harmful behaviors, including physical aggression, verbal harassment, social exclusion, rumor spreading, and cyberbullying, and is characterized by a power imbalance between perpetrator and victim [16]. Numerous cross-sectional and longitudinal studies across Western and non-Western contexts have reported that adolescents who experience peer victimization often exhibit higher levels of depressive symptoms [17,18,19,20].

Peer victimization is a pervasive phenomenon worldwide. According to the 2022 Programme for International Student Assessment (PISA), approximately 20% of students across Organisation for Economic Co-operation and Development (OECD) countries reported being bullied at least a few times a month. Among South Korean students, 8% of girls and 10% of boys reported similar experiences, both below the OECD average [21]. Despite this lower prevalence, peer victimization remains an important concern for adolescent mental health in South Korea. Even relatively mild or infrequent victimization may have meaningful developmental consequences; longitudinal evidence indicates that less intense victimization predicts subsequent increases in depressive symptoms during early adolescence [19]. For adolescents from multicultural families, such experiences may be especially consequential because they can reflect not only interpersonal rejection but also cultural marginalization and social exclusion [15,20,22].

South Korea’s cultural and educational contexts may contribute to persistent group-based peer victimization [20]. In a collectivistic culture emphasizing group harmony and conformity, victimization may often involve excluding students who deviate from group norms or display atypical physical, behavioral, or social characteristics [20]. Educational structures may reinforce this pattern because South Korean students typically remain with the same homeroom classmates throughout the academic year, while long school hours and extensive internet use may expose victimized students to repeated harassment both offline and online. In addition, despite increasing cultural diversity, the ideology of *danil minjok*, which emphasizes ethnic homogeneity, remains influential in South Korea. Adolescents from multicultural families who differ visibly, culturally, or linguistically from dominant group norms may therefore face a heightened risk of exclusion and relational aggression [22,23].

### 1.3. Acculturative Stress as a Mediator

Among adolescents from multicultural families, acculturative stress may mediate the association between peer victimization and depressive symptoms. Berry and Annis [24] introduced acculturative stress to describe immigrants’ psychological burden in adapting to the sociocultural demands of the host or dominant society. More recent approaches to acculturation have expanded the concept to encompass both the maintenance of cultural heritage and active engagement with the values and practices of the dominant culture, particularly in societies shaped by inclusive immigration policies and increasing diversity [25]. This perspective is also relevant to individuals with bicultural backgrounds, including second-generation immigrants. Acculturative stress thus refers to the psychological and physiological strain that may arise from navigating tensions between cultural heritage and dominant societal norms [25]. Within the minority stress framework, the present study conceptualizes acculturative stress as a proximal stress process reflecting adolescents’ subjective appraisals of social and cultural experiences in relation to their cultural minority status and the cognitive–affective responses arising from such appraisals. For adolescents from multicultural families, it may involve burdens associated with language-related difficulties, pressure to conform to Korean norms, teasing about their foreign-born parent’s culture of origin, and perceived discriminatory treatment linked to their multicultural family backgrounds [8,10,25,26].

Acculturative stress may intensify during adolescence, a key period for ethnic and cultural identity development [26,27], in which peer relationships play an important role [28]. Discrimination and bullying by peers from mainstream cultural backgrounds may therefore heighten acculturative stress among adolescents from immigrant or multicultural families. Research grounded in a cultural adaptation framework suggests that peer victimization is associated with greater acculturative stress among adolescents from immigrant or multicultural families [29,30]. Elevated acculturative stress has, in turn, been associated with greater depressive symptoms among adolescents from immigrant families [27,31]. Taken together, these findings suggest that acculturative stress may mediate the association between peer victimization and depressive symptoms among adolescents from multicultural families.

### 1.4. Family Support as a Moderator

Not all adolescents who experience peer victimization develop psychological symptoms. This variability can be understood through resilience and social support theories. Resilience theory emphasizes protective resources, such as family support, that help individuals cope with stressors and overcome difficulties [32]. Cohen and Wills [33] identified two mechanisms through which social support promotes well-being: a general main effect and a stress-buffering effect. Whereas the main-effect model emphasizes the overall benefits of support, the stress-buffering model proposes that support can reduce the extent to which stressors, such as peer victimization, undermine psychological health and adjustment. In early adolescence, when peer relationships become more salient but may remain emotionally volatile, family support can serve as a stabilizing resource that helps adolescents maintain self-worth, reinterpret stressful experiences, and adopt constructive coping strategies [18,33].

However, findings on whether family support mitigates peer victimization–mental health associations are mixed. Victimized youth who experience warmth and support from parents or siblings tend to report fewer depressive symptoms, with family support appearing especially protective among immigrant youth [18,34]. Family support and cohesion may also reduce stress associated with discrimination or bullying among immigrants [35,36], and supportive family relationships may buffer depressive symptoms linked to general, acculturative, or peer-related stress among adolescents [37,38,39]. Conversely, some studies have not found a buffering effect. Papafratzeskakou et al. [40] reported that family or parental support did not alter the association between peer victimization and adjustment problems among U.S. adolescents. Desjardins and Leadbeater [17] found an unexpected pattern: higher maternal emotional support was associated with greater depressive symptoms among relationally victimized youth.

### 1.5. The Present Study

The existing literature has several limitations. First, although minority stress theory was originally developed in the context of sexual minority populations [11], Meyer [11] suggested that it may apply more broadly to groups exposed to stigma, prejudice, and discrimination. Nevertheless, relatively few studies have applied this framework to culturally minoritized adolescents by examining proximal stress processes linking distal minority stressors to mental health outcomes [12,13]. Second, although peer victimization has been associated with acculturative stress among immigrant or multicultural youth [29,30], the directionality of this association remains unclear because acculturative stress may also increase vulnerability to peer victimization [41]. Prior studies have also relied largely on cross-sectional designs, sometimes using retrospective reports, limiting conclusions about temporal ordering. Applying minority stress theory to the present context, this study conceptualizes acculturative stress as a proximal process through which external stressors, such as peer victimization, may be associated with mental health difficulties. A longitudinal approach is therefore useful for examining whether peer victimization is prospectively associated with later acculturative stress and depressive symptoms, particularly because its psychological effects may persist over time [17]. Third, findings regarding the moderating role of family support have been mixed. Some studies suggest that family support buffers the effects of peer victimization or acculturative stress on depressive symptoms [34,35,36,37,38,39], whereas others have found no protective effect of family support or an unexpected amplifying effect of maternal support on the association between peer victimization and depressive symptoms [17,40]. These findings suggest that family support may operate differently across stress-related pathways rather than functioning as a uniformly protective resource.

To address these limitations, this study, guided by minority stress theory, examined whether peer victimization in the first year of middle school (T1) was prospectively associated with depressive symptoms in the second year (T2) through acculturative stress assessed at the same follow-up wave and whether family support moderated these pathways. Unlike prior MAPS-based studies focusing on immigrant mothers’ acculturative stress and parenting processes in relation to adolescents’ psychosocial adjustment [42,43,44], the present study examines adolescents’ own experiences of peer victimization and acculturative stress in relation to depressive symptoms. Acculturative stress and depressive symptoms were assessed concurrently at T2 to capture their occurrence within the same follow-up period after prior peer victimization. At T2, prior peer victimization may shape adolescents’ appraisals of social and cultural experiences in relation to their multicultural backgrounds and minority status, with these appraisals and accompanying cognitive–affective responses captured as acculturative stress and potentially associated with depressive symptoms. Although minority stress theory conceptualizes proximal stress processes as psychological mechanisms linking distal stressors to mental health outcomes [11,12], it does not specify a fixed time interval between proximal stress processes and health outcomes. Accordingly, the hypothesized pathway was examined as a theory-informed indirect association rather than definitive evidence of causal mediation. The T1–T2 period covers the first two years of middle school, when new peer relationships and sensitivity to peer evaluation become especially salient [15]. Family support was conceptualized as adolescents’ perceived family support across the T1–T2 interval, allowing the study to examine subsequent psychological responses to prior peer victimization within this support context.

**Figure 1:**
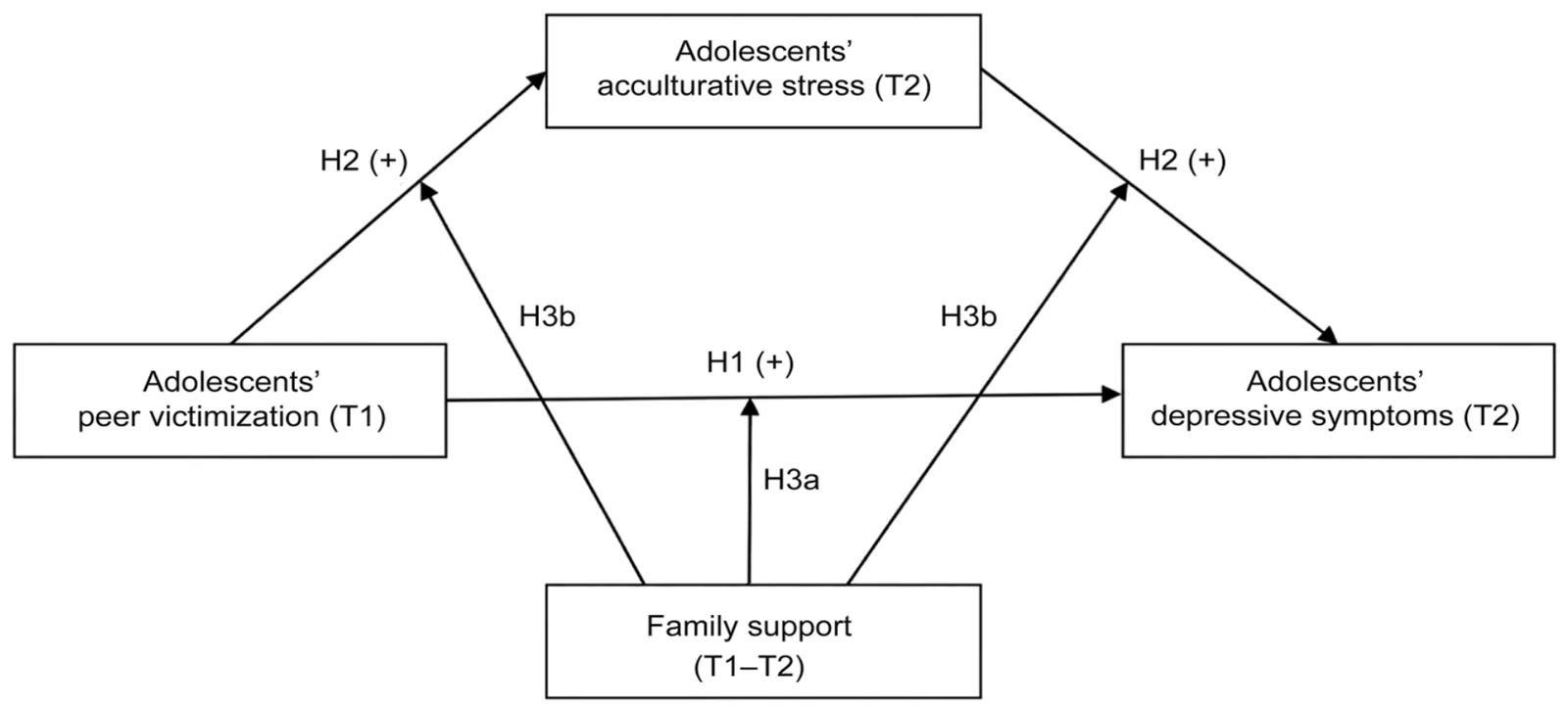
Hypothesized moderated mediation model. Note. H1–H3b correspond to Hypotheses 1, 2, 3a, and 3b. T1 = Time 1 (first year of middle school); T2 = Time 2 (second year of middle school). The signs in parentheses indicate the predicted directions of the hypothesized paths.

Based on this rationale, the following hypotheses were formulated. Peer victimization at T1 was hypothesized to predict depressive symptoms at T2 (Hypothesis 1), acculturative stress at T2 was hypothesized to mediate this association (Hypothesis 2), and family support was hypothesized to moderate both the direct association between peer victimization and depressive symptoms (Hypothesis 3a), and the indirect association between peer victimization and depressive symptoms through acculturative stress (Hypothesis 3b). Figure 1 illustrates the proposed research model.

## 2. Materials and Methods

### 2.1. Participants and Procedure

This study analyzed data from the Multicultural Adolescents Panel Study (MAPS), a nationwide longitudinal study administered by the National Youth Policy Institute (NYPI). MAPS annually follows South Korean adolescents from multicultural families and collects information on adolescents’ cultural adaptation, family and parenting contexts, school experiences, and psychosocial development [45]. For the primary analyses, the present study used Waves 4 and 5, when participants were in the first and second years of middle school, respectively. Under Korea’s 6–3–3–4 educational system (six years of elementary school, three years of middle school, three years of high school, and four years of university), these waves correspond approximately to Grades 7 and 8 in many Western educational systems.

The original MAPS cohort included 1635 adolescents in 2011; 1380 participated in Wave 4 and 1347 in Wave 5, yielding retention rates of 84.4% and 82.4%, respectively. The analytic sample included 1218 adolescents with complete data on the primary study variables across both waves (599 boys, 619 girls); thus, 162 cases were excluded from the analytic sample. To assess potential bias associated with the complete-case analytic sample, preliminary comparisons between included and excluded cases were conducted for baseline study variables. These comparisons showed no significant differences between included (*N* = 1218) and excluded cases (*n* = 162) for most study variables. Although peer victimization at T1 differed significantly between the two groups, the mean difference was small (Cohen’s |*d*| = 0.12; see Table S1 in the Supplementary Materials).

At Wave 4, adolescents had a mean age of 12.97 years (standard deviation [*SD*] = 0.35). Mothers’ and fathers’ mean ages were 43.50 years (*SD* = 5.21) and 49.22 years (*SD* = 4.55), respectively. Consistent with the typical composition of South Korean multicultural families [2,3], adolescents’ mothers were foreign-born and their fathers were South Korean. The most common maternal nationalities were Japanese (36.7%), Filipino (25.6%), ethnic Koreans from China (18.8%), and Chinese (7.0%). Among mothers, 47.0% had completed high school, 41.7% held a college degree or higher, and 11.2% had graduated from middle school; among fathers, the corresponding figures were 52.6%, 16.0%, and 31.4%, respectively. Monthly household income averaged 2.44 million Korean won (KRW; *SD* = 1.20 million) at Wave 4 and 2.51 million KRW (*SD* = 1.30 million) at Wave 5. On a four-point scale, adolescents reported high levels of self-rated Korean language proficiency at Wave 4 (mean [*M*] = 3.74, *SD* = 0.44) and Wave 5 (*M* = 3.71, *SD* = 0.48); their mothers’ Korean language proficiency was moderate to high at both Wave 4 (*M* = 3.26, *SD* = 0.55) and Wave 5 (*M* = 3.26, *SD* = 0.56).

### 2.2. Measures

#### 2.2.1. Peer Victimization

Drawing on Lee and Kim’s [46] original instrument, adolescents’ peer victimization experiences in the first year of middle school were assessed using the version adapted for MAPS. The scale comprised six items assessing the frequency of peer victimization experiences, including physical aggression, verbal abuse, rumor spreading or relational victimization, social exclusion, and mockery of physical appearance (e.g., “I have been hit, kicked, or threatened by other students”). Participants rated each item on a four-point Likert scale (1 = never to 4 = almost every day). Item-level mean scores were computed, with higher values reflecting more frequent experiences of peer victimization. The scale demonstrated excellent internal consistency (Cronbach’s α = 0.91).

#### 2.2.2. Acculturative Stress

Drawing on Hovey and King’s [47] original instrument, adolescents’ perceived acculturative stress at T2 was measured using the version adapted for MAPS. The measure included 10 items assessing stress experienced in daily life as a result of one’s cultural background (e.g., “I am looked down upon by others because my mother is a foreigner”). Although one item directly referred to peer exclusion because of having a foreign-born parent, most other items primarily assessed broader culturally specific stress related to language difficulties, cultural teasing, and perceived negative treatment of the adolescent or their family by others. Responses were rated on a four-point Likert scale (1 = not at all to 4 = very much so). Item-level mean scores were computed, with higher values indicating more severe acculturative stress. The measure was also administered at T1, and T1 scores were used for baseline comparison (see Table S1) and as a control variable in the sensitivity analysis (see Table S2). Cronbach’s α for this measure was 0.72 at T1 and 0.76 at T2, indicating acceptable internal consistency at both waves.

#### 2.2.3. Depressive Symptoms

Adolescents’ depressive symptoms at T2 were measured with the depression subscale from the Korean adaptation of the Symptom Checklist-90-Revised [48]. The scale consisted of 10 items rated on a four-point Likert scale (1 = not at all to 4 = very much so), assessing depressive symptoms such as low energy, sadness, worry, loneliness, hopelessness, and loss of interest (e.g., “I have no interest or enthusiasm in anything”). Item-level mean scores were computed, with higher values indicating more severe depressive symptoms. T1 depressive symptoms were used for baseline comparison (see Table S1) and included as a control variable in the analyses, whereas depressive symptoms assessed with the same measure at T3 were used as the outcome in the three-wave sensitivity analysis (see Table S3). Cronbach’s α was 0.92 at T1 and T2 and 0.91 at T3, indicating excellent internal consistency across all three waves.

#### 2.2.4. Family Support

The Family Support subscale of Han’s [49] Social Support Scale was administered at T1 and T2 to assess adolescents’ perceptions of family support. Item-level mean scores were first computed separately at T1 and T2 and then averaged to represent adolescents’ perceived family support across the one-year interval. The subscale consisted of seven items rated on a four-point Likert scale (1 = not at all to 4 = very much so) and reflected adolescents’ general perceptions of emotional understanding, attentive communication, mutual assistance, and caring from family members (e.g., “My family seems to understand me well”). Higher scores represented stronger perceived family support. This subscale demonstrated excellent internal consistency at both T1 and T2 (Cronbach’s α = 0.95 at both waves).

### 2.3. Statistical Analysis

Statistical analyses were conducted using IBM SPSS Statistics (Version 29.0; IBM Corp., Armonk, NY, USA). Harman’s single-factor test [50] was used as a preliminary step to explore potential common method bias stemming from adolescents’ self-report measures. Descriptive statistics for all measured variables were examined, and Pearson’s correlation coefficients were calculated to assess bivariate associations. All *p* values were two-tailed. Given the conceptual proximity and potential item-content overlap between peer victimization and acculturative stress, discriminant validity was specifically examined for peer victimization at T1 and acculturative stress at T2, which served as the predictor and mediator, respectively, in the primary analysis. Following Fornell and Larcker [51], the average variance extracted (AVE) for each construct was compared with the squared correlation between the two constructs, with AVE values exceeding the squared correlation indicating adequate discriminant validity. In addition, the concurrent correlation between peer victimization and acculturative stress at T1 was examined to assess whether the constructs remained empirically distinct when measured at the same wave.

To examine Hypotheses 1 and 2, this study utilized Model 4 of the PROCESS macro (Version 4.2) [52] in SPSS to estimate the direct association between peer victimization at T1 and depressive symptoms at T2, as well as the mediating role of acculturative stress at T2. To assess the robustness of the hypothesized indirect association, an additional sensitivity analysis was performed by re-estimating this model while controlling for baseline acculturative stress. This analysis examined whether the indirect association through acculturative stress at T2 remained significant after accounting for adolescents’ prior levels of acculturative stress. Moreover, because acculturative stress and depressive symptoms were assessed concurrently at T2 in the primary model, their temporal ordering could not be established, and reverse or reciprocal associations could not be ruled out. While the primary model was retained to examine the theory-informed pathway within the same follow-up period, an additional three-wave sensitivity analysis using peer victimization at T1, acculturative stress at T2, and depressive symptoms at T3 was conducted to examine whether the indirect association remained evident when the mediator and outcome were assessed at separate time points.

For Hypotheses 3a and 3b, which addressed the moderating roles of family support in both the direct and indirect pathways, Model 59 of the PROCESS macro was applied. To further explore the nature of these moderation effects, interaction terms were tested for three specific paths: (a) from peer victimization at T1 to depressive symptoms at T2, (b) from peer victimization at T1 to acculturative stress at T2, and (c) from acculturative stress at T2 to depressive symptoms at T2. All continuous variables involved in interaction terms were mean-centered before creating the interaction terms to reduce nonessential multicollinearity and facilitate interpretation of lower-order effects. Significant interaction effects were further probed using simple slope analyses at low (*M* − 1 *SD*) and high (*M* + 1 *SD*) levels of family support.

Indirect and conditional indirect effects were assessed using bootstrapping with 5000 resamples and 95% percentile bootstrap confidence intervals (CIs), an approach recommended for testing indirect effects because it does not assume a normal sampling distribution. Effects were considered statistically significant when the 95% CIs did not include zero. In addition, the conditional indirect effects of peer victimization on depressive symptoms through acculturative stress were examined at low (*M* − 1 *SD*) and high (*M* + 1 *SD*) levels of family support. When available in the PROCESS output, the index of moderated mediation was also examined to determine whether the indirect effect varied significantly as a function of family support.

Finally, adolescents’ sex, Korean language proficiency, and household income, which have been associated with adolescents’ depressive symptoms [53], were included as control variables in all PROCESS analyses. Specifically, Korean language proficiency and household income measured at T2, concurrently with depressive symptoms, were used in the analysis. Average monthly household income was log-transformed to reduce skewness [54]. Sex was coded as 0 for boys and 1 for girls. Baseline depressive symptoms at T1 were also controlled to address possible inflation of predictor effects due to prior outcome variance [55]. Given prior evidence of sex differences in peer victimization and depressive symptoms among adolescents [7,21,53], a supplementary analysis was conducted to examine whether sex moderated the focal pathways. In this analysis, sex was entered as the moderator rather than as a control variable.

A total of 12 cases (0.98%) had missing values, all of which were limited to covariates. These values were imputed using sample mean substitution prior to analysis. Because missing data were restricted to covariates and affected fewer than 1% of cases, the impact of this approach on the focal estimates is expected to be minimal [56]. Consistent with this expectation, an additional analysis using listwise deletion (*n* = 1206) yielded substantively identical results in terms of coefficient magnitudes and statistical significance.

## 3. Results

### 3.1. Preliminary Analysis

An assessment of common method bias using Harman’s single-factor test showed that the first factor explained 26.06% of the variance, which is below the 50% threshold, suggesting that common method bias was unlikely to be a major concern in the present study [50]. Table 1 reports the means, standard deviations, and intercorrelations for the primary study variables. Peer victimization at T1 was positively correlated with acculturative stress and depressive symptoms measured at T2. Acculturative stress at T2 was also positively correlated with depressive symptoms, whereas family support was negatively correlated with peer victimization, acculturative stress, and depressive symptoms.

Given the potential overlap of peer victimization and acculturative stress, their discriminant validity was examined more closely. Their prospective correlation was small (*r* = 0.10), and the AVE values for peer victimization (0.65) and acculturative stress (0.59) exceeded their squared correlation (*r*^2^ = 0.01), supporting discriminant validity. The concurrent correlation between peer victimization and acculturative stress at T1 was also small (*r* = 0.18, *p* < 0.001), further indicating that the two constructs were related but not redundant.

### 3.2. Acculturative Stress as a Mediator Between Peer Victimization and Depressive Symptoms

To examine Hypotheses 1 and 2, this study tested whether peer victimization at T1 was associated with depressive symptoms at T2 both directly and indirectly via acculturative stress at T2. As shown in Table 2, peer victimization at T1 was significantly associated with both acculturative stress (*B* = 0.12, *p* < 0.01) and depressive symptoms (*B* = 0.15, *p* < 0.01) at T2, after adjusting for household income, sex, Korean language proficiency, and baseline depressive symptoms, supporting Hypothesis 1. When acculturative stress was included in the model with depressive symptoms as the outcome, both peer victimization (*B* = 0.11, *p* < 0.05) and acculturative stress (*B* = 0.35, *p* < 0.001) remained significantly associated with depressive symptoms. The estimated indirect effect was significant (*B* = 0.04, 95% percentile bootstrap CI [0.002, 0.10]), supporting Hypothesis 2 regarding the hypothesized indirect association.

To account for the potential influence of adolescents’ preexisting acculturative stress on the hypothesized indirect pathway, a sensitivity analysis was conducted by additionally including baseline acculturative stress as a control variable (see Table S2). The estimated indirect effect remained significant (*B* = 0.07, 95% percentile bootstrap CI [0.003, 0.14]), whereas the direct association between peer victimization and depressive symptoms was attenuated to non-significance. This pattern provides additional support for the robustness of the indirect association through acculturative stress.

In addition, the three-wave sensitivity analysis showed that peer victimization at T1 was significantly associated with acculturative stress at T2 (*B* = 0.12, *p* < 0.01), which in turn was significantly associated with depressive symptoms at T3 (*B* = 0.17, *p* < 0.001). The estimated indirect effect was also significant (*B* = 0.02, 95% percentile bootstrap CI [0.0004, 0.05]), although the association between acculturative stress and depressive symptoms was smaller than in the primary model using depressive symptoms at T2 (see Table S3).

### 3.3. Family Support as a Moderator in the Pathways Linking Peer Victimization to Depressive Symptoms Through Acculturative Stress

To test Hypotheses 3a and 3b, this study used PROCESS Model 59, controlling for household income, sex, Korean language proficiency, and baseline depressive symptoms. As shown in Table 3, peer victimization at T1 was significantly associated with acculturative stress (*B* = 0.08, *p* < 0.05) and depressive symptoms (*B* = 0.12, *p* < 0.05) at T2. Acculturative stress at T2 was also significantly associated with depressive symptoms (T2; *B* = 0.38, *p* < 0.001). Family support was negatively associated with both acculturative stress (T2; *B* = −0.18, *p* < 0.001) and depressive symptoms (T2; *B* = −0.29, *p* < 0.001). Family support significantly moderated the association between peer victimization (T1) and acculturative stress (T2; *B* = −0.15, *p* < 0.01) and the association between acculturative stress (T2) and depressive symptoms (T2; *B* = 0.26, *p* < 0.001). However, family support did not moderate the direct association between peer victimization (T1) and depressive symptoms (T2; *B* = −0.09, *p* = 0.17); therefore, Hypothesis 3a was not supported.

**Figure 2:**
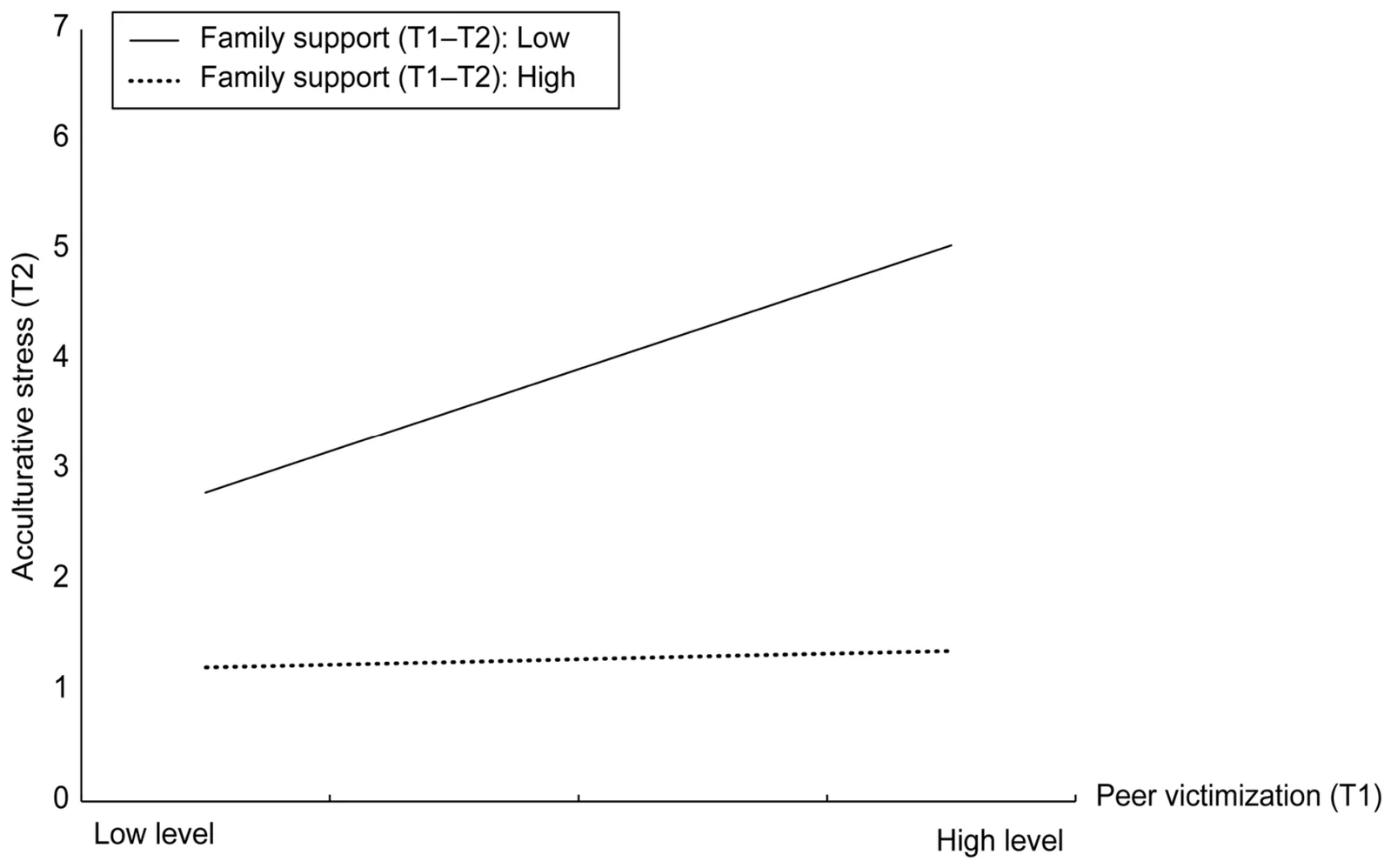
Interaction of peer victimization (T1) and family support (T1–T2) predicting acculturative stress (T2). Note. *N* = 1218. T1 = Time 1 (first year of middle school); T2 = Time 2 (second year of middle school). Predicted values are plotted to illustrate the interaction pattern; simple slopes correspond to the regression coefficients reported in Table 3.

**Figure 3:**
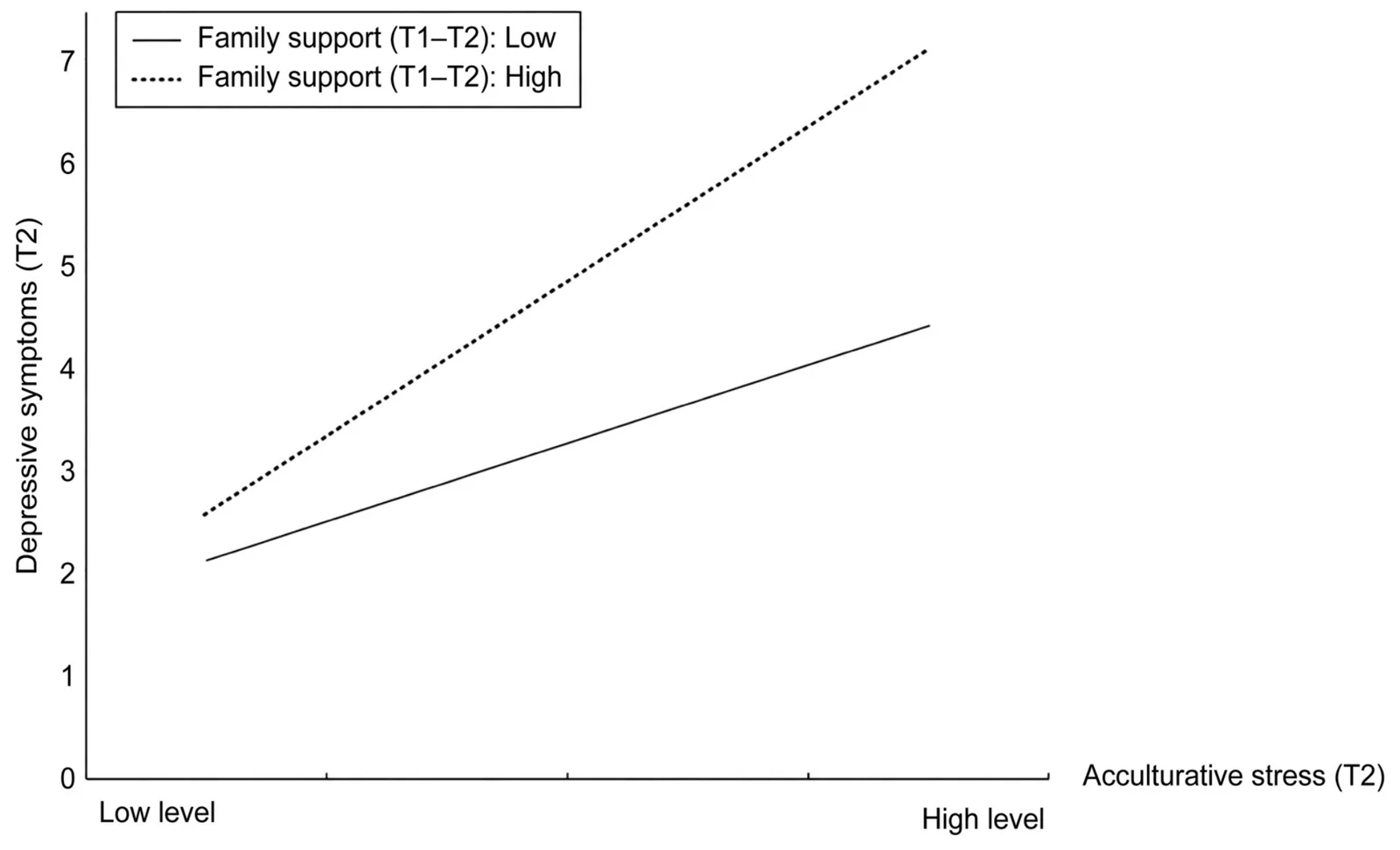
Interaction of acculturative stress (T2) and family support (T1–T2) predicting depressive symptoms (T2). Note. *N* = 1218. T1 = Time 1 (first year of middle school); T2 = Time 2 (second year of middle school). Predicted values are plotted to illustrate the interaction pattern; simple slopes correspond to the regression coefficients reported in Table 3.

As illustrated in Figure 2, follow-up simple slope analyses indicated that peer victimization (T1) was significantly associated with higher acculturative stress (T2) when family support was low (*M* − 1 *SD*; *B* = 0.16, *p* < 0.001) but not when it was high (*M* + 1 *SD*; *B* = 0.01, *p* = 0.831). As depicted in Figure 3, the association between acculturative stress and depressive symptoms at T2 was significant at both low (*M* − 1 *SD*; *B* = 0.25, *p* < 0.001) and high (*M* + 1 *SD*; *B* = 0.50, *p* < 0.001) levels of family support, with a stronger association at high levels of family support. Taken together, higher levels of family support weakened the positive association between peer victimization and acculturative stress but strengthened the positive association between acculturative stress and depressive symptoms.

**Figure 4:**
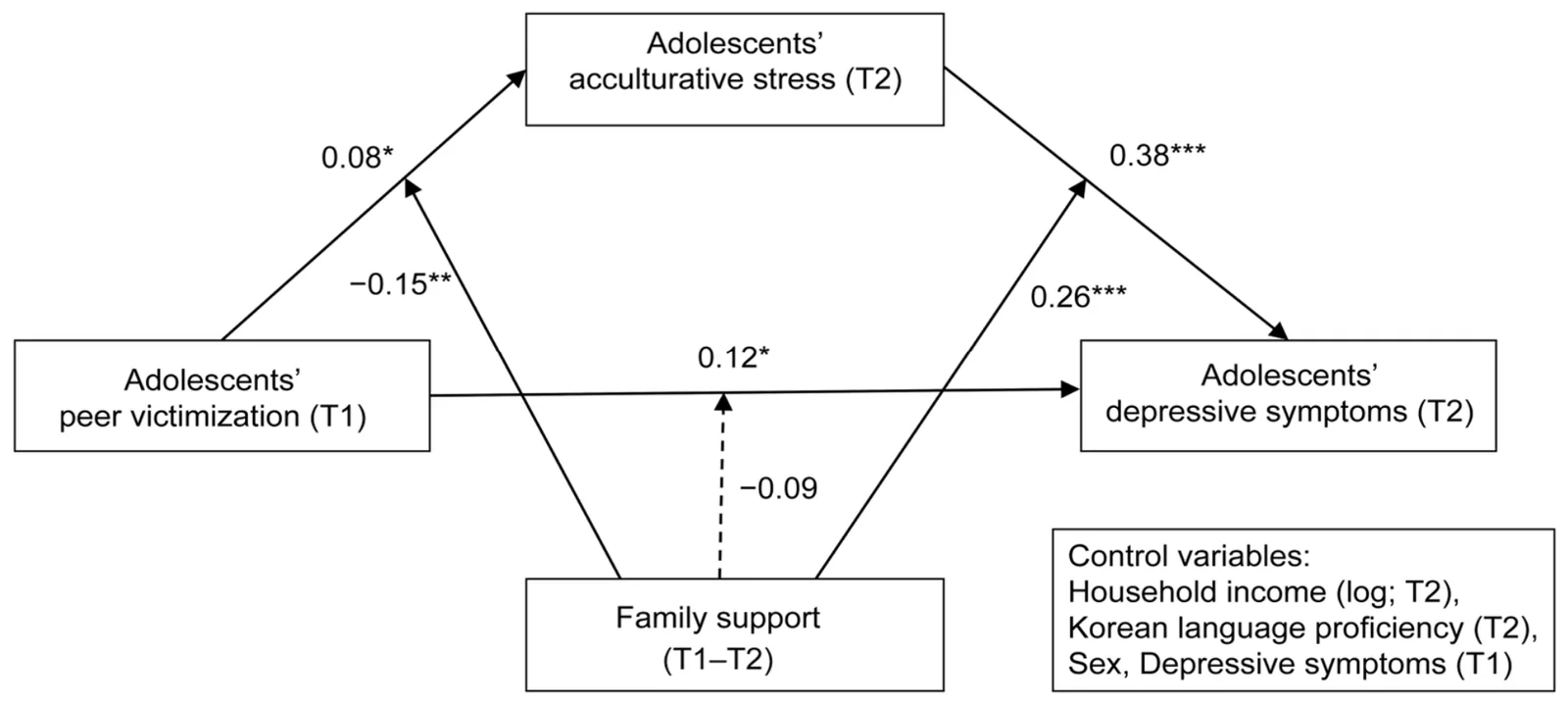
Tested moderated mediation model. Note. *N* = 1218. T1 = Time 1 (first year of middle school); T2 = Time 2 (second year of middle school); log = log-transformed. The values on the paths indicate unstandardized path coefficients. Solid arrows indicate significant paths; the dashed arrow indicates a nonsignificant path. * *p* < 0.05. ** *p* < 0.01. *** *p* < 0.001.

In addition, conditional indirect effects were estimated at each level of family support using 5000 bootstrap resamples and 95% percentile bootstrap CIs. The conditional indirect effects of peer victimization on depressive symptoms through acculturative stress were not statistically significant at either low (*B* = 0.03, 95% percentile bootstrap CI [−0.003, 0.10]) or high (*B* = 0.01, 95% percentile bootstrap CI [−0.07, 0.05]) levels of family support. These results reflect what has been described in the moderated mediation literature as competing (or offsetting) moderation effects [52]: family support weakened the peer victimization–acculturative stress path but strengthened the acculturative stress–depressive symptoms path, such that the two path-level moderations did not aggregate into a single overall moderated mediation effect on the indirect pathway. Consistent with this pattern, PROCESS did not generate a single index of moderated mediation for this model. Thus, Hypothesis 3b received partial support: family support significantly moderated each of the two component paths of the indirect association (peer victimization → acculturative stress and acculturative stress → depressive symptoms), although these effects operated in opposing directions and the conditional indirect effects through acculturative stress did not reach statistical significance. Figure 4 depicts the tested moderated mediation model.

In a supplementary analysis, sex significantly moderated the association between peer victimization at T1 and acculturative stress at T2 (*B* = −0.18, *p* < 0.05). Peer victimization was positively associated with acculturative stress among boys (*B* = 0.21, *p* < 0.001), whereas the corresponding association among girls was not statistically significant (*B* = 0.04, *p* = 0.52). The other interaction effects involving sex were not significant (Table S4).

## 4. Discussion

Grounded in minority stress theory, this study examined how peer victimization, acculturative stress, depressive symptoms, and family support were linked across the early middle-school period among South Korean multicultural adolescents.

The prospective association between peer victimization and depressive symptoms underscores the psychological significance of peer victimization during early adolescence, consistent with prior longitudinal findings in youth [19,57]. Although the reported frequency of peer victimization was low in the present sample, such levels are not uncommon in community-based, nonclinical youth samples [19,58]. Importantly, recent longitudinal evidence indicates that even less intense peer victimization is associated with subsequent increases in depressive symptoms during early adolescence [19]. Thus, the present findings suggest that relatively infrequent peer victimization may still pose meaningful psychological risks for adolescents from multicultural families, including higher levels of depressive symptoms and acculturative stress.

The indirect association through acculturative stress, together with its persistence after controlling for baseline acculturative stress at T1, is consistent with minority stress theory [11]. This framework conceptualizes proximal stress processes as psychological mechanisms linking distal stressors with mental health outcomes. However, because acculturative stress and depressive symptoms were both assessed at T2, the primary indirect association should be interpreted as reflecting a theory-informed psychological pathway rather than as definitive evidence of causal mediation. This conceptualization of temporally proximal psychological processes is also supported by daily diary research on minority stress, which suggests that daily minority stress experiences and emotional distress may be closely linked over relatively short time frames [59]. Using a longitudinal design, this study extends prior cross-sectional or retrospective work [29,30,41] by providing prospective evidence that peer victimization may precede heightened acculturative stress. Because adolescence is a key period for cultural and ethnic identity development, peers may play an important role in shaping how youth understand and negotiate their cultural backgrounds [26,27,28]. Furthermore, the association between acculturative stress and depressive symptoms is consistent with prior research on culturally minoritized adolescents [27,31]. Related research in a different adolescent population suggests that adverse interpersonal experiences may shape later adjustment through maladaptive schemas involving beliefs and expectations about oneself, others, and interpersonal relationships [60]. In the present context, peer victimization may likewise heighten negative appraisals and emotional responses related to adolescents’ own cultural backgrounds and their relationships with peers from the dominant cultural group, thereby contributing to acculturative stress and potentially to depressive symptoms.

To further examine the hypothesized temporal ordering, an additional three-wave sensitivity analysis was conducted using peer victimization at T1, acculturative stress at T2, and depressive symptoms at T3. The significant indirect association in this temporally separated model provided complementary support for the proposed sequence. However, the model cannot rule out residual confounding by unmeasured child-, family-, or school-related factors. Future longitudinal research should therefore examine the proposed pathway while accounting for additional factors associated with adolescent depressive symptoms, including school-related stressors and other relevant child and family factors.

Family support may not function as a uniformly protective resource but may instead operate differently depending on the stage of the stress process. Family support attenuated the association between peer victimization at T1 and acculturative stress at T2, consistent with Cohen and Wills’ [33] stress-buffering framework and minority stress theory, which emphasizes the protective role of contextual support in reducing stress responses to minority-related disadvantage [14]. This finding also aligns with prior evidence that social support can reduce discrimination-related or acculturative stress among culturally minoritized groups [35,36]. In South Korea, many multicultural adolescents who experience peer victimization seek help from their parents [10], and family members’ emotional support and understanding—grounded in a shared multicultural family context—may help adolescents make sense of these peer experiences without attributing them primarily to their cultural backgrounds or minority status, thereby attenuating the extent to which peer victimization translates into acculturative stress.

In contrast, higher levels of family support strengthened the positive association between acculturative stress and depressive symptoms. This unexpected pattern suggests that family support may not operate as a uniformly protective factor across different stages of the stress process. One possibility for future investigation is whether family discussions of distressing experiences sometimes involve processes resembling co-rumination, in which repeated discussions of problems and negative emotions foster emotional closeness but provide limited problem-solving [61]. In this context, repeated conversations about peer victimization or acculturative stress may be associated with heightened depressive symptoms rather than constructive coping [17]. This interpretation is also consistent with evidence that rumination—an intrapersonal process involving persistent focus on problems or negative affect, conceptually related to co-rumination but occurring at the individual level—can mediate the association between minority stressors and depression [62].

Another possibility concerns how the form and timing of family support may shape adolescents’ responses at different stages of the stress process. During the early stage of stress appraisal (peer victimization → acculturative stress), family support may function as a stabilizing resource. By contrast, at later stages, when stress becomes more internalized (acculturative stress → depressive symptoms), the association with depressive symptoms may be stronger at higher levels of family support, particularly when certain forms of support are less well aligned with adolescents’ emotional needs. Processes such as co-ruminative family communication, passive or avoidant coping, maladaptive emotion-regulation processes (e.g., rumination), and repeated discussion that does not adequately meet adolescents’ emotional needs may warrant consideration as possible contributors to the stronger association observed at higher levels of family support. However, because these processes were not directly assessed in the present study, they should be treated as hypotheses for future research rather than as explanations supported by the present data.

The Korean family and school context may also be relevant to understanding this pattern. In South Korea, mothers typically assume primary caregiving responsibilities [63]. Moreover, formal responses to peer victimization are often handled through school-led administrative procedures in which parents may play relatively limited roles [64]. These contextual features may be particularly salient for multicultural families, where immigrant mothers may face language barriers and have limited familiarity with the Korean school system. However, the extent to which these factors shaped family communication, parental responses, or the observed moderation pattern was not examined in the present study. Accordingly, future research should examine whether immigrant mothers’ Korean-language proficiency and familiarity with the Korean education system influence family responses, adolescents’ coping, and adjustment in the context of peer victimization and acculturative stress.

This conflicting role of family support reflects a pattern described in the moderated mediation literature as competing (or offsetting) moderation effects [52], which may help explain why the conditional indirect effects were not significant and why the index of moderated mediation was not generated. Specifically, the two significant moderation effects operated in opposite directions: family support weakened the peer victimization → acculturative stress path but strengthened the acculturative stress → depressive symptoms path. Within Hayes’s conditional process framework, the index of moderated mediation reflects whether moderation of the component paths combines to produce an overall moderation of the indirect effect [52,65]. When these path-specific moderation effects operate in opposite directions, they can offset each other, making the overall moderated mediation pattern difficult to interpret.

Furthermore, unlike prior research [18,34], this study found no significant evidence that family support moderated the direct pathway from peer victimization to depressive symptoms. This finding does not necessarily imply that social support is ineffective in this context. Rather, it suggests that support systems more proximal to the school context—such as peers, teachers, school counselors, or other professionals—may be especially important in mitigating depressive symptoms associated with peer victimization. Given that school occupies a large part of adolescents’ daily lives, future research should examine how school-based and professional support shape the pathway from peer victimization to depressive symptoms and subsequent adjustment outcomes.

### 4.1. Strengths and Limitations

From a theoretical perspective, the present study contributes to minority stress theory by extending its application to multicultural adolescents in South Korea. Specifically, by identifying a pattern of associations consistent with the distal-to-proximal stress process posited by minority stress theory—linking an external peer experience, an internalized culturally specific stress response, and broader mental health difficulties—this study supports the relevance of the theory for understanding mental health risks among culturally minoritized adolescents. It also extends social support and stress-buffering perspectives, which have emphasized the protective role of support in the stress process [14,33], by showing that perceived family support may not function as a uniformly protective context but rather as a context-dependent factor whose role varies across stress-related pathways.

Although this study yielded important contributions, certain limitations should be acknowledged. First, because this study focused on family support, it could not directly compare the roles of different sources of support, such as family, peers, teachers, school counselors, or other professionals. This variability is evident in prior research: Wasserman et al. [31] reported that peer support, but not parental support, moderated the association between acculturative stress and depression among U.S. Latinx adolescents, whereas Shah et al. [34] identified family cohesion and school belonging as protective factors among immigrant-background youth who experienced bullying. Future research should therefore examine how family, peer, school, and professional support systems differentially shape pathways from peer victimization to psychosocial outcomes through acculturative stress. Second, although the additional three-wave sensitivity analysis strengthened the examination of temporal ordering, the primary mediation and moderated mediation models relied on two time points, with acculturative stress and depressive symptoms assessed concurrently at T2. This design limits causal inference, and reciprocal or alternative temporal processes cannot be ruled out. Future multi-wave longitudinal studies should measure peer victimization, acculturative stress, and depressive symptoms at separate time points and, ideally, repeatedly assess these constructs and control for prior levels of both the mediator and the outcome to more rigorously evaluate temporal ordering and reciprocal associations.

Third, the generalizability of the findings should be considered in relation to the characteristics of the MAPS analytic sample. Because the MAPS panel was established in 2011, the analytic sample reflects the composition of adolescents recruited into and retained in the MAPS panel and may not fully reflect the current demographic composition of multicultural families in South Korea. In this sample, Japanese, Filipino, and ethnic Korean Chinese mothers were the most prevalent maternal nationalities, whereas more recent national statistics indicate that Vietnamese and Chinese mothers now constitute the largest groups among foreign-born mothers in multicultural families [4]. Therefore, future research using data that better reflect the changing demographic composition of South Korean multicultural families would help assess the generalizability of the present findings. In addition, studies involving adolescents from immigrant and multicultural families across diverse cultural contexts, family structures, parental origins, and ethnic backgrounds, as well as cross-cultural comparisons, are needed to determine whether the observed mechanisms operate similarly across populations.

Fourth, some measurement limitations should be noted. The internal consistency of the acculturative stress measure at T1 was acceptable but relatively modest (Cronbach’s α = 0.72). Because T1 acculturative stress was included as a control variable in the sensitivity analysis, its relatively modest internal consistency may have affected the precision of that supplementary analysis. Future research should employ more reliable and culturally validated measures of acculturative stress. Relatedly, family support was assessed as a broad, unidimensional perception of support from family members and did not distinguish among specific dimensions of support or between mother- and father-specific support. In addition, averaging the T1 and T2 scores captured the overall level of support rather than changes in support over time. Therefore, future research should use multidimensional and parent-specific measures and examine changes in family support over time.

Finally, the study relied on adolescents’ self-report measures for key constructs, raising potential concerns about common method bias. Although Harman’s single-factor test did not indicate a serious concern, shared method variance cannot be entirely ruled out. Future research would benefit from incorporating multi-informant data, such as reports from parents, teachers, or peers, or combining self-report measures with observational or administrative data.

### 4.2. Practical Implications and Conclusions

The findings support coordinated school- and family-based responses. Schools should foster culturally inclusive environments through culturally responsive multicultural education and anti-bullying initiatives. They should also establish clear procedures for safely reporting peer victimization and facilitate the early identification of adolescents experiencing peer victimization, acculturative stress, or depressive symptoms. In particular, anti-bullying programs should encourage bystanders to avoid reinforcing bullying through laughter or online sharing and to support victimized peers safely. School counseling systems should also provide or arrange access to professionals with expertise in acculturation, cultural adjustment, and adolescent mental health. At the family level, parents may benefit from guidance on recognizing adolescents’ distress, maintaining open communication, and encouraging constructive coping and problem-solving.

Grounded in minority stress theory, this study extends the framework to South Korean adolescents from multicultural families using a longitudinal approach that remains relatively uncommon in this population. The findings provide prospective evidence consistent with a distal-to-proximal stress process in which peer victimization is associated with later acculturative stress, which in turn is linked to depressive symptoms. Because acculturative stress and depressive symptoms were assessed concurrently at T2, this indirect pathway should be interpreted as a theory-informed longitudinal association rather than as definitive evidence of causal mediation. The study also identifies a complex and potentially opposing role of family support: family support weakened the association between peer victimization and acculturative stress but strengthened the association between acculturative stress and depressive symptoms. Overall, the findings broaden the application of minority stress theory to a culturally minoritized adolescent population in South Korea and highlight the importance of considering both peer-related minority stress and the complex role of family support in adolescent mental health.

## Figures and Tables

**Table 1:** Descriptive summary of key variables, including means, standard deviations, and correlation coefficients.

Key Variables	1.	2.	3.	4.
1. PV (T1)				
2. AS (T2)	0.10 **			
3. DEP (T2)	0.16 **	0.30 **		
4. FS (T1–T2)	−0.10 **	−0.25 **	−0.42 **	
*M*	1.05	1.45	1.69	3.20
*SD*	0.26	0.37	0.54	0.48

Note. *N* = 1218. PV = peer victimization; AS = acculturative stress; DEP = depressive symptoms; FS = family support; T1 = Time 1 (first year of middle school); T2 = Time 2 (second year of middle school); *M* = mean; *SD* = standard deviation. All scale scores are based on item-level means. ** *p* < 0.01.

**Table 2:** Mediation analysis of acculturative stress (T2) in the association between peer victimization (T1) and depressive symptoms (T2).

Outcome	Predictors	*B*	*SE*	*t*	*R* ^2^	*F*
AS (T2)	HI (log)	0.01	0.02	0.35	0.03	8.01 ***
	Sex	−0.03	0.02	−1.30		
	KL	−0.08	0.02	−3.69 ***		
	dep (T1)	0.06	0.02	3.03 **		
	PV (T1)	0.12	0.04	2.79 **		
DEP (T2)	HI (log)	0.02	0.03	0.68	0.21	63.40 ***
	Sex	0.11	0.03	3.93 ***		
	KL	−0.12	0.03	−4.01 ***		
	dep (T1)	0.36	0.02	14.84 ***		
	PV (T1)	0.15	0.05	2.80 **		
DEP (T2)	HI (log)	0.02	0.03	0.61	0.26	72.67 ***
	Sex	0.12	0.03	4.43 ***		
	KL	−0.09	0.03	−3.11 **		
	dep (T1)	0.34	0.02	14.51 ***		
	PV (T1)	0.11	0.05	2.12 *		
	AS (T2)	0.35	0.04	9.73 ***		

Note. *N* = 1218. AS = acculturative stress; HI (log) = household income (log-transformed); KL = Korean language proficiency; dep = baseline depressive symptoms (control variable at Time 1); PV = peer victimization; DEP = depressive symptoms; T1 = Time 1 (first year of middle school); T2 = Time 2 (second year of middle school); *B* = unstandardized coefficient; *SE* = standard error. Sex was entered as a dichotomous variable, with boys coded as 0 and girls coded as 1. * *p* < 0.05. ** *p* < 0.01. *** *p* < 0.001.

**Table 3:** Moderation analysis of family support (T1–T2) in the associations among peer victimization (T1), acculturative stress (T2), and depressive symptoms (T2).

Outcome	Predictors	*B*	*SE*	*t*	*R* ^2^	*F*
AS (T2)	HI (log)	0.01	0.02	0.61	0.08	15.61 ***
	Sex	−0.03	0.02	−1.35		
	KL	−0.06	0.02	−2.68 **		
	dep (T1)	0.00	0.02	0.21		
	PV (T1)	0.08	0.04	1.97 *		
	FS (T1–T2)	−0.18	0.02	−7.54 ***		
	PV (T1) × FS (T1–T2)	−0.15	0.06	−2.60 **		
DEP (T2)	HI (log)	0.03	0.03	1.03	0.33	67.11 ***
	Sex	0.11	0.03	4.44 ***		
	KL	−0.05	0.03	−1.76		
	dep (T1)	0.26	0.02	10.61 ***		
	PV (T1)	0.12	0.05	2.22 *		
	AS (T2)	0.38	0.04	9.47 ***		
	FS (T1–T2)	−0.29	0.03	−10.02 ***		
	PV (T1) × FS (T1–T2)	−0.09	0.07	−1.38		
	AS (T2) × FS (T1–T2)	0.26	0.05	5.48 ***		

Note. *N* = 1218. AS = acculturative stress; HI (log) = household income (log-transformed); KL = Korean language proficiency; dep = baseline depressive symptoms (control variable at Time 1); PV = peer victimization; FS = family support; DEP = depressive symptoms; T1 = Time 1 (first year of middle school); T2 = Time 2 (second year of middle school); *B* = unstandardized coefficient; *SE* = standard error. Interaction terms involving family support, denoted by × and computed as products of the relevant mean-centered variables, were included for the PV (T1) → AS (T2), AS (T2) → DEP (T2), and PV (T1) → DEP (T2) paths. Sex was entered as a dichotomous variable, with boys coded as 0 and girls coded as 1. * *p* < 0.05. ** *p* < 0.01. *** *p* < 0.001.

## Data Availability

The data presented in this study are available from the National Youth Policy Institute at https://www.nypi.re.kr/archive/mps/program/examinDataCode/dataDwloadAgreeView?menuId=MENU00226 (accessed on 12 October 2025). These data were derived from the Multicultural Adolescents Panel Study, a publicly available research resource.

## Supplementary Materials

The following supporting information can be downloaded at: https://www.mdpi.com/article/10.3390/children13081052/s1. Table S1: Comparison of included and excluded adolescents based on baseline study variables; Table S2: Sensitivity analysis of the mediation model controlling for baseline acculturative stress; Table S3: Three-wave sensitivity analysis of the mediation model using peer victimization at T1, acculturative stress at T2, and depressive symptoms at T3; Table S4: Moderating effects of sex on the associations among peer victimization (T1), acculturative stress (T2), and depressive symptoms (T2).

## Abbreviations

The following abbreviations are used in this manuscript:

AS	Acculturative stress
FS	Family support
CI	Confidence interval
DEP	Depressive symptoms
HI	Household income
KL	Korean language proficiency
KRW	Korean won
*M*	Mean
MAPS	Multicultural Adolescents Panel Study
NYPI	National Youth Policy Institute
OECD	Organisation for Economic Co-operation and Development
PISA	Programme for International Student Assessment
PV	Peer victimization
*SD*	Standard deviation
*SE*	Standard error
SPSS	Statistical Package for the Social Sciences
T1	Time 1
T2	Time 2
T3	Time 3
