# Supplementary material for: From Peer Victimization to Depressive Symptoms Through Acculturative Stress in South Korean Multicultural Adolescents: The Complex Moderating Roles of Family Support
Source: Children (Basel). 2026 Aug 6;13(8):1052. doi: 10.3390/children13081052 (PMC13511416; doi:10.3390/children13081052)
Supplement: Supplementary file 1 [file children-13-01052-s001.zip › children-4453057-supplementary.pdf]

Table S1. Comparison of included and excluded adolescents based on baseline study variables.

Variable	Excluded (n = 162) M (SD)	Included (N = 1218) M (SD)	p
KL	3.75 (0.41)	3.74 (0.44)	0.361
PV (T1)	1.03 (0.12)	1.05 (0.26)	0.018
DEP (T1)	1.64 (0.51)	1.64 (0.54)	0.466
AS (T1)	1.42 (0.37)	1.38 (0.31)	0.090
Sex	N/A	N/A	0.490

Note. KL = Korean language proficiency; PV = peer victimization; DEP = depressive symptoms; AS = acculturative stress; T1 = Time 1 (first year of middle school). Participants who did not provide complete data on the study variables were excluded from the analytic sample. Although PV (T1) differed significantly between the included and excluded groups, the effect size (Cohen's $|d| = 0.12$) was small. Independent-samples t tests were used for continuous variables, and chi-square tests were used for sex ($\chi^2(1) = 0.48$, $p = 0.490$). The sex distribution was 53.7% female in the excluded group and 50.8% in the included group.

Table S2. Sensitivity analysis of the mediation model controlling for baseline acculturative stress.

Outcome	Predictors	*B*	*SE*	*t*	R^2	*F*
AS (T2)	HI (log)	0.01	0.02	0.41	0.07	15.08***
	Sex	−0.03	0.02	−1.38		
	KL	−0.07	0.02	−3.16**		
	dep (T1)	0.02	0.02	1.19		
	as (T1)	0.17	0.04	4.85***		
	PV (T1)	0.20	0.04	5.27***		
DEP (T2)	HI (log)	0.02	0.03	0.68	0.22	55.29***
	Sex	0.11	0.03	3.89***		
	KL	−0.11	0.03	−3.67***		
	dep (T1)	0.34	0.03	13.51***		
	as (T1)	0.15	0.05	3.11**		
	PV (T1)	0.14	0.05	2.91**		
DEP (T2)	HI (log)	0.02	0.03	0.60	0.27	62.60***
	Sex	0.12	0.03	4.38***		
	KL	−0.08	0.03	−2.96**		
	dep (T1)	0.33	0.02	13.64***		
	as (T1)	0.09	0.05	1.92		
	PV (T1)	0.08	0.05	1.61		
	AS (T2)	0.33	0.04	9.15***		

Note. $N = 1218$. AS = acculturative stress; HI (log) = household income (log-transformed); KL = Korean language proficiency; dep = baseline depressive symptoms (control variable at Time 1); as = baseline acculturative stress (control variable at Time 1); PV = peer victimization; DEP = depressive symptoms; T1 = Time 1 (first year of middle school); T2 = Time 2 (second year of middle school); *B* = unstandardized coefficient; *SE* = standard error. Sex was entered as a dichotomous variable, with boys coded as 0 and girls coded as 1. The analysis also yielded a significant indirect effect ($B = 0.07$, 95% percentile bootstrap CI [0.003, 0.14]), as the CI excluded zero. ** $p < 0.01$. *** $p < 0.001$.

Table S3. Three-wave sensitivity analysis of the mediation model using peer victimization at T1, acculturative stress at T2, and depressive symptoms at T3.

Outcome	Predictors	*B*	*SE*	*t*	R^2	*F*
AS (T2)	HI (log)	0.01	0.02	0.22	0.03	7.78***
	Sex	−0.03	0.02	−1.22		
	KL	−0.08	0.02	−3.68***		
	dep (T1)	0.06	0.02	2.93**		
	PV (T1)	0.12	0.04	2.78**		
DEP (T3)	HI (log)	0.05	0.03	1.56	0.15	42.63***
	Sex	0.10	0.03	3.60***		
	KL	−0.06	0.03	−1.85		
	dep (T1)	0.32	0.03	12.54***		
	PV (T1)	0.12	0.06	2.13*		
DEP (T3)	HI (log)	0.05	0.03	1.55	0.17	39.19***
	Sex	0.11	0.03	3.78***		
	KL	−0.04	0.03	−1.39		
	dep (T1)	0.31	0.03	12.23***		
	PV (T1)	0.10	0.06	1.79		
	AS (T2)	0.17	0.04	4.34***		

Note. *N* = 1196. AS = acculturative stress; HI (log) = household income (log-transformed); KL = Korean language proficiency; dep = baseline depressive symptoms (control variable at Time 1); PV = peer victimization; DEP = depressive symptoms; T1 = Time 1 (first year of middle school); T2 = Time 2 (second year of middle school); T3 = Time 3 (third year of middle school); *B* = unstandardized coefficient; *SE* = standard error. Sex was entered as a dichotomous variable, with boys coded as 0 and girls coded as 1. The analysis also yielded a significant indirect effect (*B* = 0.02, 95% percentile bootstrap CI [0.0004, 0.05]), as the CI excluded zero. $^{*}p < 0.05$. $^{**}p < 0.01$. $^{***}p < 0.001$.

Table S4. Moderating effects of sex on the associations among peer victimization (T1), acculturative stress (T2), and depressive symptoms (T2).

Outcome	Predictors	*B*	*SE*	*t*	R^2	*F*
AS (T2)	HI (log)	0.01	0.02	0.43	0.04	7.43***
	KL	−0.08	0.02	−3.60***		
	dep (T1)	0.06	0.02	2.93**		
	PV (T1)	0.21	0.06	3.45***		
	Sex	−0.03	0.02	−1.28		
	PV (T1) × Sex	−0.18	0.08	−2.10*		
DEP (T2)	HI (log)	0.02	0.03	0.62	0.27	54.53***
	KL	−0.09	0.03	−3.10**		
	dep (T1)	0.34	0.02	14.49***		
	PV (T1)	0.12	0.08	1.56		
	AS (T2)	0.37	0.05	7.75***		
	Sex	0.12	0.03	4.43***		
	PV (T1) × Sex	−0.02	0.10	−0.19		
	AS (T2) × Sex	−0.06	0.07	−0.80		

Note. $N = 1218$. AS = acculturative stress; HI (log) = household income (log-transformed); KL = Korean language proficiency; dep = baseline depressive symptoms (control variable at Time 1); PV = peer victimization; DEP = depressive symptoms; T1 = Time 1 (first year of middle school); T2 = Time 2 (second year of middle school); *B* = unstandardized coefficient; *SE* = standard error. Interaction terms involving sex, denoted by ×, were included for the PV (T1) → AS (T2), AS (T2) → DEP (T2), and PV (T1) → DEP (T2) paths. The continuous predictors were mean-centered before the interaction terms were computed. The significant PV (T1) × Sex interaction indicated that peer victimization at T1 was positively associated with acculturative stress at T2 among boys ($B = 0.21$, $p < 0.001$), whereas this association was not statistically significant among girls ($B = 0.04$, $p = 0.52$). Sex was entered as a dichotomous variable, with boys coded as 0 and girls coded as 1. $^{*}p < 0.05$. $^{**}p < 0.01$. $^{***}p < 0.001$.
